# Sequence-based information-theoretic features for gene essentiality prediction

**DOI:** 10.1186/s12859-017-1884-5

**Published:** 2017-11-09

**Authors:** Dawit Nigatu, Patrick Sobetzko, Malik Yousef, Werner Henkel

**Affiliations:** 1 Transmission Systems Group, Jacobs University Bremen, Campus Ring 1, Bremen, D-28759 Germany; 2 Philipps-Universität Marburg, LOEWE-Zentrum für Synthetische Mikrobiologie, Hans-Meerwein-Straße, Mehrzweckgebäude, Marburg, 35043 Germany; 3 Community Information Systems, Zefat Academic College, Zefat, 13206 Israel

**Keywords:** Essential genes, Random Forest, Information-theoretic features, Machine learning

## Abstract

**Background:**

Identification of essential genes is not only useful for our understanding of the minimal gene set required for cellular life but also aids the identification of novel drug targets in pathogens. In this work, we present a simple and effective gene essentiality prediction method using information-theoretic features that are derived exclusively from the gene sequences.

**Results:**

We developed a Random Forest classifier and performed an extensive model performance evaluation among and within 15 selected bacteria. In intra-organism predictions, where training and testing sets are taken from the same organism, AUC (Area Under the Curve) scores ranging from 0.73 to 0.90, 0.84 on average, were obtained. Cross-organism predictions using 5-fold cross-validation, pairwise, leave-one-species-out, leave-one-taxon-out, and cross-taxon yielded average AUC scores of 0.88, 0.75, 0.80, 0.82, and 0.78, respectively. To further show the applicability of our method in other domains of life, we predicted the essential genes of the yeast *Schizosaccharomyces pombe* and obtained a similar accuracy (AUC 0.84).

**Conclusions:**

The proposed method enables a simple and reliable identification of essential genes without searching in databases for orthologs and demanding further experimental data such as network topology and gene-expression.

**Electronic supplementary material:**

The online version of this article (doi:10.1186/s12859-017-1884-5) contains supplementary material, which is available to authorized users.

## Background

The subset of genes which are necessary for the viability and reproduction of an organism are called essential genes. Detection of these genes is very crucial for understanding the minimal requirements for maintaining life [1, 2]. Since the disruption or deletion of essential genes of a pathogen results in the death of the organism, essential genes can be used as potential drug targets [3, 4]. Furthermore, studies on essential genes are very important in synthetic biology for re-engineering microorganisms and creating cells with a minimal genome [5].

Genome-wide systematic or random experimental laboratory procedures such as transposon mutagenesis [6], single gene knock-out [7, 8], and RNA interference [9] are used to identify the EGs. Although the experimental methods are fairly accurate, they are often time-consuming and expensive. Moreover, gene essentiality results of the experimental methods may depend on growth conditions [10]. To bypass these constraints, various computational prediction methods have been proposed. The earliest computational methods were based on comparative genomics in which gene essentiality annotations are transferred among species through homology mappings [11, 12]. Later on, as the list of genes for model organisms became available in public databases (such as DEG [13], CEG [14], and OGEE [15]), researchers have studied the characteristics and features of essential genes and deployed machine-learning based prediction methods.

A wide range of features has been associated with gene essentiality. The features can be broadly categorized into sequence information (e.g., GC content, protein length, and codon composition) [16–18], network topology (e.g., degree centrality and clustering coefficient) [19–22], homology (e.g., number of paralogs) [17, 23, 24], gene expression (e.g., mRNA expression level and fluctuations in gene-expression) [22, 25], cellular localization (e.g., cytoplasmic score and outer membrane score) [22, 26, 27], functional domain (e.g., domain enrichment) [25], and physicochemical property (e.g., molecular weight and number of moles of amino acids) [26, 27].

Except for the sequence based and sequence derived features, which can be obtained directly from the DNA or protein sequences, the others require pre-computed experimental data. Network topology based features require the availability or construction of protein-protein interaction, gene regulatory networks, or metabolic networks. Similarly, the gene expression and functional domain features demand the expression data and a search in protein domain databases such as PROSITE and PFAM. Although experimental and genetic network information is available for the well-studied species, they are not available for all organisms, especially not for the newly sequenced and under-studied. Hence, predictors relying only on sequence information are of special importance.

A number of researchers have proposed sequence-based essential gene predictors [16–18, 23, 26–29]. Ning et al. [16] used nucleotide, di-nucleotide, codon, and amino acid frequencies along with what is known as CodonW features. The CodonW features, which are sequence derived, are obtained from a codon usage analysis software (http://codonw.sourceforge.net). However, some of the CodonW features are not purely obtainable from the DNA or protein sequence. For instance, the Codon Adaptation Index (CAI) is a measure of the relative adaptability of the codon usage of a gene compared to the codon usage of highly expressed genes [30]. That means, one needs to first distinguish the highly expressed genes in the organism. Due to its effectiveness, the CAI feature is used by all sequence based predictors. Ning et al. performed cross-validation experiments considering 16 bacteria species. The other very effective essential gene predictor based solely on sequence and sequence-derived properties is Song et al’s ZUPLS [17]. ZUPLS uses features from the so-called Z-curve, sequence-based (e.g., size, CAI, and strand), homology mapping, and domain enrichment scores. Cross-organism results were shown using models trained on *E. coli* and *B. subtilis*. Among the sequence based methods, ZUPLUS is the best method. Although homology and domain information are sequence based, they require a priori information in databases. In 2011, Palaniappan and Palaniappan [26] presented a predictor based on sequence, pysio-chemical properties, and cellular localization information. In addition to predictions of essential genes between organisms (leave-one-species-out and cross-validation), they showed results at a higher taxonomic level (leave-one-taxon-out). Very recently, Liu et al. [27] using similar features to [26] made an extensive study on 31 bacteria species and presented self-test, cross-validation, pairwise, and leave-one-species-out experimental results. Yu et al. [18] and Li et al. [28] used a different set of features based on fractal and inter-nucleotide distance sequences. In 2013, a method called Geptop (gene essentiality prediction tool based on orthology and phylogeny) [23] was proposed and due to the high accuracy and the availability of a Web server, it is the most used computational tool. Geptop identifies orthologs by the reciprocal best hit method and computes evolutionary distance between genomes using the Composition Vector (CV) method [31]. Then, an essentiality score is defined and a threshold-based classification is performed.

Other computational methods which use sequence information together with network topology and gene expression include the works of Deng et al. [25] and Cheng et al. [22, 24]. Deng et al. [25] have used thirteen features. Along with the sequence dependent features such as protein length and number of codons, they have used features related to network topology, gene-expression, homology, phylogenetics, and protein domain knowledge. A combination of four machine-learning algorithms (Naïve Bayes, logistic regression, C4.5 decision tree, and CN2 rule) were applied. They showed the effective transferability of essentiality annotations among *E. coli, B. subtilis, Acinetobacter baylyi,* and *Pseudomonas aeruginosa*. Cheng et al. [22] proposed a novel computational method which is based on Naive Bayes classifier, logistic regression, and a genetic algorithm. They have used a combination of network topology, gene expression, and sequence-related features and reciprocally predicted essential genes among 21 species. To our knowledge, Cheng et al.’s predictor is the best, in terms of higher prediction accuracy.

In a previous work [32], we proposed a support vector machine (SVM) based predictor using information-theoretic features and relying only on sequence information and showed that decent results can be obtained. However, most of the analysis was limited to very few commonly used bacteria. The information-theoretic features are entropy (Shannon and Gibbs), mutual information (MI), conditional mutual information (CMI), and Markov model based. These quantities measure the structural and organizational properties in the DNA sequences. The entropy computations will highlight the degree of randomness and thermodynamic stability of the genes. In [33], we have analyzed the application and implication of Shannon and Gibbs entropies in bacterial genomes. MI has been extensively used in various computational biology and bioinformatics applications. For instance, MI profiles were used as genomic signatures to reveal phylogenetic relationships between genomic sequences [34], as a metric of phylogenetic profile similarity [35], and for identification of single nucleotide polymorphisms (SNPs) [36]. Hence, MI and CMI features make use of sequence organization and dependencies and capture the differences between essential and non-essential genes. The Markov features are selected for measuring statistical dependencies.

In the present work, in addition to the information-theoretic features used in [32], Kullback-Leibler divergence (KLD) between the distribution of k-mers (*k*=1,2,3) in the genes and the corresponding distributions in the organism used for training, total CMI, total MI, and 2 more entropy features were included. Moreover, we used a Random Forest classifier and executed an extensive model evaluation within and among 15 bacteria species. To show the applicability of our method to in other domains of life, essential genes of the fission yeast *Schizosaccharomyces pombe* were predicted. Moreover, in addition to the common evaluation approaches such as cross-validation in a single organism, pairwise cross-organism predictions, and leave-one-species-out, to assess the generalization performance of our models, following the approach pointed out in [26], we performed cross-taxon and leave-one-taxon-out experiments. The obtained results are then compared to the 8 pre-existing prediction methods mentioned above.

## Methods

### Data sources

The essential and non-essential protein coding genes for the 16 species were obtained from the database of essential genes (DEG 13.5). DEG collects the list of essential genes in both eukaryotes and prokaryotes, which were identified by various gene knock-out experimental procedures such as transposon mutagenessis and RNA interference [13]. The list of species used in this study is presented in Table 1. The genome sequences were downloaded from the NCBI database (ftp://ftp.ncbi.nih.gov/genomes/).

**Table 1:** The list and detail of the organisms used in this work

No.	Organism	Abbr.	Number of essential genes	Number of non-essential genes	Accession No.
1	Acinetobacter baylyi ADP1	AB	499	2594	NC_005966
2	Bacillus subtilis 168	BS	271	3904	NC_000964
3	Escherichia coli MG1655	EC	296	4077	NC_000913
4	*Francisella novicida U112*	FN	*392*	*1329*	NC_008601
5	*Haemophilus influenzae Rd KW20*	HI	*642*	*512*	NC_000907
6	*Helicobacter pylori 26695*	HP	*323*	*1135*	NC_000915
7	Mycoplasma genitalium G37	MG	381	94	NC_000908
8	Mycoplasma pulmonis UAB CTIP	MP	310	322	NC_002771
9	*Mycobacterium tuberculosis H37Rv*	MT	*614*	*2552*	NC_000962
10	Pseudomonas aeruginosa UCBPP-PA14	PA	335	960	NC_008463
11	Staphylococcus aureus N315	SA	302	2281	NC_002745
12	*Staphylococcus aureus NCTC 8325*	*SA2*	*351*	*2541*	*NC_007795*
13	Salmonella enterica serovar Typhi	SE	353	4005	NC_004631
14	Salmonella typhimurium LT2	ST	230	4228	NC_003197
15	*Vibrio cholerae N16961*	VC	*779*	*2943*	NC_002505
16	Schizosaccharomyces pombe 972h-	SP	1260	3573	NC_003424

### Information theoretic features

In computational biology and bioinformatics, information-theoretic quantities have been widely used to model, analyze, and/or measure both structural and organizational properties in biological sequences. In this work, we used IT quantities to produce features which will enable the classification of essential and non-essential genes. The features used in this study are: 4 entropy (E), 17 mutual information (MI), 65 conditional mutual information (CMI), 3 Kullback-Leibler divergence (KLD), and 2 Markov model (M) related. Here, we present a brief description of the information-theoretic quantities used in this work, which was also presented in [32]. A detailed description can be found in standard information theory text books [37].

#### Mutual information (MI)

The mutual information measures the information shared by two random variables. It is the amount of information provided by one random variable about the other. Here, mutual information was used to measure the information between consecutive bases *X* and *Y* and is mathematically defined as

1 $$ I(X,Y) = \sum_{x \in \Omega}\sum_{y \in \Omega}P(x,y) \log_{2} \frac{P(x,y)}{P(x)P(y)}\;,  $$

where *Ω* is the set of nucleotides {*A,T,C,G*}, *P*(*x,y*) is the joint probability, and *P*(*x*) and *P*(*y*) are the marginal probabilities. The probabilities are estimated from their relative frequencies in the corresponding gene sequences. Along with the total mutual information computed according to Eq. (1), for each base pair (*x,y*), the quantity $P(x,y) \log _{2} \frac {P(x,y)}{P(x)P(y)}$ is calculated and used as a feature. Therefore, a total of 17 MI-related features were calculated.

#### Conditional mutual information (CMI)

The mutual information between two random variables *X* and *Y* conditioned on a third random variable *Z* having a probability mass function (pmf) *P*(*z*) is given by

2 $$ {\begin{aligned} I(X;Y|Z) &= \sum_{z \in \Omega} P(z)\sum_{x \in \Omega}\sum_{y \in \Omega}P(x,y|z) \log_{2} \frac{P(x,y|z)}{P(x|z)P(y|z)} \\ & =\sum_{x \in \Omega}\sum_{y \in \Omega}\sum_{z \in \Omega}P(x,y,z) \log_{2} \frac{P(z)P(x,y,z)}{P(x,z)P(y,z)} \end{aligned}}  $$

where *P*(*x,yz*), *P*(*x,z*), and *P*(*y,z*) are the joint pmfs of the random variables shown in brackets. The three positions in a DNA triplet are regarded as the random variables X, Z, and Y, respectively. The mutual information between the bases at the first and the third position conditioned on the base in the middle is calculated according to Eq. (2) and used as a feature. In addition, for each possible triplet, the quantity $P(x,y,z) \log _{2} \frac {P(z)P(x,y,z)}{P(x,z)P(y,z)}$ was calculated. Resulting in a total of 65 CMI-based features.

#### Entropy (E)

The Shannon entropy [38] quantifies the average information content of the gene sequence from the distribution of symbols. The Shannon entropy for a block size of *N* is defined as

3 $$ H_{N}=-\sum_{i}P_{s}^{(N)}(i) \log_{2} P_{s}^{(N)}(i)\;,  $$

where $P_{s}^{(N)}(i)$ is the probability of the *i* ^*th*^ word of block size *N*. Shannon entropies of the genes were calculated for block sizes of 2 and 3.

Similarly, the Gibbs entropy is defined as

4 $$ S_{G}=-k_{B}\sum_{i}P^{N}_{G}(i) \ln P^{N}_{G}(i)\;,  $$

where *P*_*G*_(*i*) is the probability to be in the *i* ^*th*^ state and *k*_*B*_ is the Boltzmann constant (1.38×10^−23^ J/K). Gibbs’ entropy is similar to Shannon’s entropy except for the Boltzmann constant. Nevertheless, unlike the Shannon case, where the probability is defined according to the frequency of occurrence, we associated the probability distribution with the thermodynamic stability quantified by the nearest-neighbor free energy parameters. The probability distribution, *P*_*G*_(*i*), is modeled by the Boltzmann distribution given by

5 $$ P^{N}_{G}(i)=\frac{n_{i}e^{-\frac{E(i)}{k_{B}T}}}{\sum\limits_{j}{n_{j}e^{-\frac{E(j)}{k_{B}T}}}}\;.  $$

*n*_*i*_ is the frequency of the *i* ^*th*^ word of block size *N* and *T* is the temperature in Kelvin. *E*(*i*) is the energy of the codon according to [39]. For block sizes greater than two, the energies were computed by adding the involved di-nucleotides. Shannon and Gibbs entropies for block size of 2 and 3 were calculated and used as features.

#### Markov (M)

Assuming that the gene sequences in the essential and non-essential classes are generated by two separate Markov sources, we construct a Markov chain and use the scores of the genes as Markov features. The training set is subdivided into a subset containing the essential and non-essential samples. Thereafter, each subset is used to generate a Markov chain of a preselected or estimated order *m* (*MC*_+_(*m*) and *MC*_−_(*m*) for essential and non-essential genes, respectively). The transition probabilities of the two Markov chains are empirically estimated using the so-called Lidstone estimator [40, 41]. Let *N*_*x*_(*v*) denote the number of times a word *v* of length *m* appears in a training sequence *x*. The probability that the next nucleotide is *a*, where *a*∈*Ω*={*A,C,G,T*}, conditioned on the context *v*∈*Ω* ^*m*^ is

6 $$ P_{v,a}=\frac{N_{x}(va) + \delta}{N_{x}(v) + 4\delta}\;.  $$

The parameter *δ* assigns a pseudo count to unseen symbols to avoid zero probabilities. We experimentally checked and found that the smaller values of *δ* are better and consequently set *δ*=0.001. After the two Markov chains were constructed, they were used to score each gene sequence.

First, the correct Markov chain order for both EGs and NEGs in the training dataset is estimated. Then, two Markov chains of the estimated orders are constructed. After that, the features are computed by scoring every gene using the generated Markov chains. If we represent the sequence as *b*_1_,*b*_2_,*b*_3_,...,*b*_*L*_, the score is calculated as

7 $$ \begin{aligned} Score &= \sum\limits_{i=1}^{L-m}P\left(b_{i}b_{i+1} \dots b_{i+m}\right)\\ & \quad \log_{2} \left(\frac{P\left(b_{i+m}|b_{i}b_{i+1} \dots b_{i+m-1}\right)}{P(b_{i+m})}\right)\;. \end{aligned}  $$

The score gives an indication of how likely the sequence is generated by the given *m*-th order Markov chain. The scores of the gene sequence on the Markov chains *MC*_+_(*m*) and *MC*_−_(*m*) were used as features. For inter-organism essentiality predictions, the Markov orders were estimated from the training sets. As shown in [32], the estimated order provided better results. After evaluating the performances of selected Markov order estimators in the literature [41–45], the CMI based estimator proposed by Papapetrou and Kugiumtzis [46] is chosen. However, in cross-organism and cross-taxa predictions, order estimation increased the computational complexity without improving the result. Hence, we decided to use a fixed order Markov chain. After experimenting with orders 1 up to 6, order 1 (i.e., *m*=1) was selected.

#### Kullback-Leibler divergence (KLD)

The Kullback-Leibler divergence (KLD) [47] measures the similarity of a probability distribution *P*(*x*) to a model distribution *Q*(*x*), and it is calculated as

8 $$ KLD=\sum_{i}P(x) \log_{2} \frac{P(x)}{Q(x)}\;.  $$

The frequencies of the nucleotides, di-nucleotides, and tri-nucleotides in a given gene sequence were compared against the corresponding frequencies in the genome of the organism used for training the model (background distributions).

### Classifier design and evaluation

Feature preparation and computations were performed using Python 3.5.2. We implemented a Random Forest classifier using the data analytics platform Konstanz Information Miner (KNIME 3.3.1) [48]. Information gain is used as a split criteria. Typically, the number of non-essential genes is significantly larger than that of the essential genes. To balance the two classes, various schemes of under- and over-sampling approaches could be taken. Since it was shown in [18] that the choice of a balancing approach does not influence the performance of essential gene predictions, we selected the random under-sampling of non-essential genes.

In cross-organism predictions, classifiers were trained on one (or more) organism and tested on another, whereas in intra-organism predictions 80% of the data is used for training the models and 20% is used for testing. The random selections were repeated 100 times, i.e., 100-fold Monte Carlo cross-validation were performed for model establishment.

The Area Under the Curve (AUC) of the Receiver Operating characteristic Curve (ROC) was used to evaluate the performance of our classifier. The ROC plots the true positive rate versus false positive rate. It shows the trade-off between sensitivity and specificity for all possible thresholds. Other performance evaluation such as F-measure and Accuracy were also calculated. However, these parameters depend on the selected threshold value. Therefore, we mainly used the AUC score for analyzing the performance of the classifier. The evaluation of our model using the other measures can be obtained from the the provided Additional file.

## Results and discussion

### Intra-organism cross-validation predictions

**Fig. 1:**
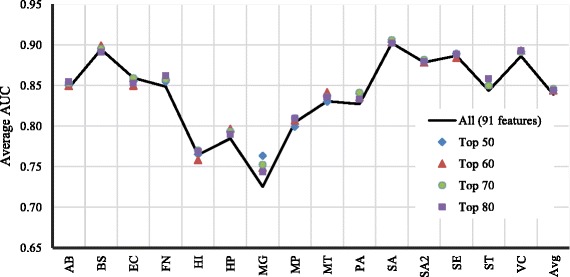
Average AUC scores of intra-organism essential gene predictions in 15 bacteria species. The prediction performance of the top 50,60,70, and 80 features based on information gain is also shown

In intra-organism predictions, both the training and testing data is obtained from the same organism. The average AUC scores of a 100-fold Monte Carlo cross-validation experiment on the 15 bacteria are presented in Fig. 1. The values range between 0.73 and 0.90, 0.84 on average. Except for three bacteria, namely HI, HP, and MG, the AUC scores are more than 0.80. We also performed a feature selection experiment using the information gain rankings, selecting the top 50, 60, 70, and 80 features (Fig. 1). Using the top 70 features provided the better accuracy on average. For MG taking only the top 50 features yielded a 4% gain. The result demonstrates that fewer features can be used to improve the computational complexity without affecting the accuracy of the predictions. Overall, the improvement gained by feature selection is not significant. Therefore, in the remaining parts of this work, feature selection is not considered. To assess the contributions of the different feature types to the classification task, the information gain rankings for all species were collected and a global feature ranking was obtained (Additional file 1: Table S1). The top 20 features consists of 8 MI, 8 CMI, 2 entropy, 1 Markov, and 1 KLD features. This shows that all feature classes contribute to the high prediction performances.

Compared to Ning et al. [16] essentiality predictor which uses only sequence based and sequence derived features, our method yielded better AUC scores. The AUC scores for EC and MP were improved from 0.82 to 0.86 and from 0.74 to 0.80, respectively. Similarly, in comparison with the inter-nucleotide distance sequences based essential gene predictor proposed by Li et al. [28], our method provided an improvement of up to 9%. For EC, the AUC score is improved from 0.80 to 0.86, for BS from 0.81 to 0.89, for SE from 0.80 to 0.89, and for SA from 0.88 to 0.90. In addition, our average AUC score (0.84) was also much better than Yu et al. [18] fractal features based predictor (0.77 on 27 selected bacteria).

### Cross-organism predictions

So far, both the training and test sets were taken from a single genome. In this section, models trained on a given organism (or groups) are used to predict the essential and non-essential genes of another distinct organism. Cross-organism predictions are more realistic and useful in *ab initio* identification of essential genes. Two approaches were taken. The first approach is a pairwise cross-organism prediction in which models trained on one species are used to predict the essential and non-essential genes of every other species, separately. The other approach is a leave-one-species-out procedure whereby genes of the 14 bacteria are collectively used for establishing a model and essential genes of the remaining bacterium are predicted.

#### Pairwise predictions

**Fig. 2:**
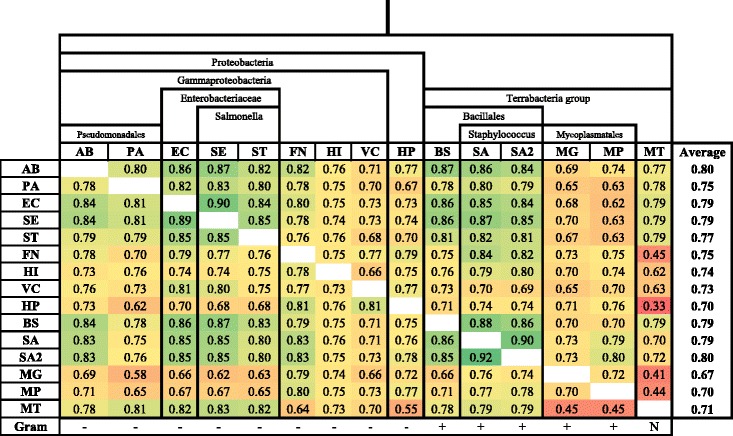
Pairwise cross-organism predictions results. 15×15 average AUC scores are presented. The phylogenetic relationship and the taxonomic classification of the bacteria are also shown

Pairwise cross-organism predictions among the 15 bacteria were performed to see how well essentiality annotations can be transferred between both closely and distantly related species. A heat map of the 21×21 average AUC matrix is presented in Fig. 2. The bacteria are also grouped together according to the phylogenetic tree constructed using the PhyloT tree generator (http://phylot.biobyte.de/index.html). The overall prediction performances were very good (AUC scores of up to 0.92 were obtained). However, cross-predictions among MT and MG, MP, FN, and HP are very bad, even sometimes worse than a random guess. As described in [12, 22], larger evolutionary distance, differences in growth conditions, phenotypes, and lifestyles, and poor quality of the training data may have led to poor performances.

Although close evolutionary distance and similar lifestyles provide common essential gene characteristics, the results for the distantly related species were also good. For instance, BS and EC diverged over a billion years ago [49], before the divergence of plants and animals, and yet highly accurate predictions were possible (AUC score of 0.86). In addition, models trained based on the taxonomic orders Bacillales (BS, SA, SA2) and Enterobacterales (EC, SE, ST) produced better overall performance. Hence, future blind essentiality predictions of a new species can be done using one of these bacteria.

The performance of our predictor is as good as the other existing state-of-the art gene essentiality predictors which use homology, gene-expression and network topology based features in addition to sequence-derived information. Note that sequence similarity searching is computationally expensive. The comparison to Deng et al. [25] and Song et al. [17] ZUPLS classifiers among AB, BS, EC, and PA is shown in Table 2. On average, our method is slightly better than Deng et al’s (2%). ZUPLS is the best method among the sequence-based predictors and on average it is only 3% better than our method. However, since a database search for homology and domain information are not required, our method could be more advantageous in case of limited computational power.

**Table 2:** Comparing prediction performance (average AUC score) among AB, BS, EC and PA

Train	Test	Deng et al. [25]	Song et al. [17]	Our method
AB	EC	0.89	0.91	0.86
BS	AB	-	0.86	0.84
BS	EC	0.86	0.91	0.86
BS	PA	-	0.81	0.78
EC	AB	0.8	0.86	0.84
EC	BS	0.8	0.93	0.86
EC	PA	-	0.81	0.81
PA	EC	0.82	-	0.82
**Average**		**0.83**	**0.87**	**0.84**

Cheng et al. [24] and Liu et al. [27] made pairwise predictions on 21 and 31 species, respectively, providing the 21×21 and 31×31 AUC matrices. We filtered out the common bacterial species and compared the results. Here, it should be noted that, in all the three methods, the classifiers for each species are trained independently and tested on every other species. Hence, taking the sub-group (15×15) and comparing the results is fair. Looking at the distribution of the AUC scores and the corresponding mean AUC values, our predictor (0.75) was 14% better than Liu et al.’s (0.61) while Cheng et al.’s predictor (0.79), being the best essentiality predictor, was 4% better than ours. Considering that Cheng et al. used network, gene expression, and homology information, the AUC scores of our method are very good.

#### Leave-one-species-out predictions

In the leave-one-species-out approach, we predicted the essential/non-essential genes of one species using a model trained on the remaining 14 bacterial annotated genes. This approach is also very practical for blind essentiality annotations of new organisms. In [32], we performed this analysis using an SVM classifier. Here, the Random Forest machine learning algorithm is used, alternatively.

The prediction performance of our method using both SVM and Random Forest classifiers is shown in Table 3. Apart from MG whose AUC score is 0.68, very good results (AUC ≥ 0.75) were obtained for all other species. Both machine learning algorithms yielded a similar 0.8 average AUC score and comparable results on individual species. This shows that the high prediction accuracy of our method is due to the ability of the information-theoretic features to capture gene essentiality/non-essentiality attributes.

**Table 3:** Leave-one-species-out results using SVM and Random Forest classifiers

	Our method		Liu et al.	Palaniappan and Mukherjee	Geptop (homology)	Geptop* (Composition)
Training on (No. of species)	14		30	14	18	18
	Random Forest	SVM	SVM	SVM	Score based	Score based
AB	0.81	0.83	0.75	0.74	0.85	0.79
BS	0.84	0.84	0.77	0.58	0.95	0.81
EC	0.87	0.88	0.83	0.65	0.95	0.84
FN	0.83	0.83	0.67	0.66	0.84	0.74
HI	0.75	0.77	0.54	0.46	0.57	0.59
HP	0.75	0.74	0.52	0.59	0.60	0.64
MG	0.68	0.66	0.60	0.64	0.72	0.56
MP	0.75	0.74	0.64	0.61	0.87	0.76
MT	0.80	0.77	0.70	0.49	0.73	0.77
PA	0.80	0.80	0.65	0.66	0.80	0.79
SA	0.88	0.90	0.81	0.66	0.84	0.86
SA2	0.86	0.85	0.80	-	0.88	0.83
SE	0.86	0.86	0.69	-	0.95	0.86
ST	0.81	0.79	0.84	0.53	0.71	0.69
VC	0.75	0.72	0.69	-	0.61	0.72
**Average**	**0.80**	**0.80**	**0.70**	**0.61**	**0.79**	**0.75**

The average AUC scores of four existing methods are also presented for comparison. Geptop* is a sequence composition based predictor presented along with Geptop [23]

Three studies have used a leave-one-species-out approach to assess the performance of their models. Palaniappan and Mukherjee [26] in 2011, Geptop [23] in 2013, and Liu et al. [27] in 2017. The average AUC score has a 10% and 19% improvement over Liu et al.’s and Palaniappan and Mukherjee’s, respectively. Our method is also comparable to Geptop. However, for well-studied organisms like EC and BS, Geptop is significantly better. Along with the homology- and phylogeny-based predictor, in [23], the results of another method, called integrative compositional information predictor, were reported. Codon and amino acid compositions and CodonW features (158 features) were used. Compared to this method which used sequence composition features, our method is slightly better.

#### Cross-validation on all bacteria

The other most common method to asses the prediction accuracy of machine learning models is a 5-fold cross-validation. After the total data consisting of 6078 essential genes and 33477 non-essential genes is divided into 5 separate folds, each fold is tested on a model trained on the combination of the other 4 folds. Average AUC score of 0.88 was obtained. Again, in comparison with Ning et al. [16] (0.82 AUC) and Palaniappan and Mukherjee [26] (0.8 AUC), our method is superior.

### Cross-taxonomic predictions

**Fig. 3:**
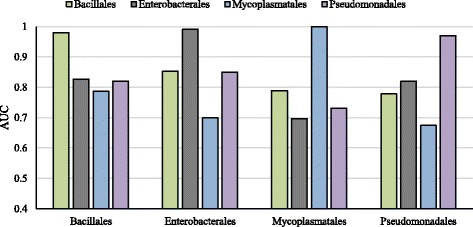
Cross-taxon prediction results

**Fig. 4:**
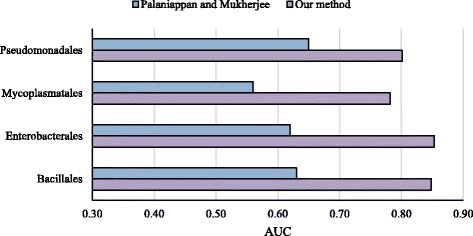
Leave-one-taxon out predictions of our method and an existing method [26]

Palaniappan and Mukherjee [26] tested the generalization ability of their classifiers across taxonomic boundaries. We made a similar assessment on our classifier at higher taxonomic level. Species belonging to a similar taxonomic order are grouped together (see Fig. 2) and cross-taxon and leave-one-taxon-out tests were made. The four taxonomic orders are Bacillales (BS, SA, and SA2), Enterobacterales (EC, SE, and ST), Mycoplasmatales (MG and MP), and Pseudomonadales (AB and PA). Species without a taxonomic pair were left out of this taxonomic analysis. The cross-taxonomic results are depicted in Fig. 3. The cross-taxonomic results are as good as the cross-organism counterparts. For example, the prediction of EC using BS yielded 0.86 AUC score and predicting Enterobacterales using Bacillales also yielded 0.85. In the leave-one-taxon-out setting, very accurate results were obtained. For Bacillales and Enterobacterales the average AUC scores were 0.85 whereas Mycoplasmatales and Pseudomonadales had 0.78 and 0.80, respectively. In comparison to Palaniappan and Mukherjee our classifier produced an outstanding performance (Fig. 4).

### Essential gene prediction of an eukaryotic organism

**Fig. 5:**
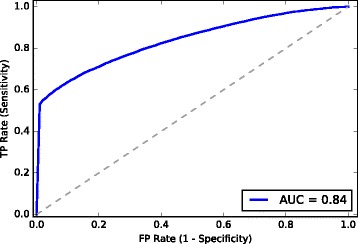
ROC curve for the prediction of *Schizosaccharomyces pombe* essential genes

To verify the applicability of our method to the prediction of essential genes in other domains of life, we selected the fission yeast *Schizosaccharomyces pombe* which is regarded as a very important model organism for the study of eukaryotic molecular and cellular biology [50]. It has 1260 essential and 3573 non-essential genes. The Random Forest classifier was trained using 80% of the data and is tested on the remaining 20%, performing 50-fold Monte Carlo cross-validation steps. The average ROC curve is shown in Fig. 5. An average AUC score of 0.84 was obtained, which is consistent with the prediction results of the bacterial genomes. This shows that information-theoretic measures can be reliably used for the prediction of essential genes also across all domains of life. We also tested the transferability of essentiality annotations from bacteria to yeast. A model trained on the 15 bacteria was used for classification and a relatively low AUC score of 0.65 was obtained. Classifiers trained on EC and BS yielded better AUC scores of 0.76 and 0.79, respectively. The reason for the low cross-organism prediction performance and the detailed application of the proposed method on eukaryotic organisms shall be investigated in a future work.

## Conclusions

We proposed a machine-learning based computational method for predicting essential genes using information-theoretic measures as features. The features are directly derived from the DNA sequence and hence can be applied to any species. The applicability of the existing computational methods which make use of network topology, gene ontology annotations, and gene-expression depends on the availability of pre-computed experimental data such as protein/gene interaction networks and gene-expression data. However, these experimental data are available for a few well-studied organisms. Other works of gene essentiality predictions also use homology and functional domain knowledge through database searches. Although the homology features are sequence-based, the computational complexity for sequence alignment is very high. Therefore, our method provides a simple and reliable alternative.

Extensive performance evaluation using different setups were performed on selected 15 bacterial species. In intra-organism predictions, very high AUC scores ranging from 0.73 to 0.9 were obtained. In cross-organism pairwise predictions, the vast majority of the results are very good. Scores as high as 0.92 and mean AUC of 0.75 were achieved. However, due to factors such as high evolutionary distance, different lifestyles, growth conditions, and phenotypes there were very few poor results [25]. Based on the results, for future blind predictions, we suggest using one of the well-studied bacteria, such as *B. subtilis* and *E. coli* (the essentiality annotations are of high quality). In addition, 5-fold cross-validation and leave-one-species-out experiments have yielded average AUC scores of 0.88 and 0.80, respectively. Furthermore, our model performed very well at higher taxonomic ranks (order). An average score of 0.82 in cross-taxon and 0.78 in leave-one-taxon-out predictions, which is significantly superior to the previously published result having average AUC of 0.62. Finally, in order to show that our method is not limited to essential gene prediction of bacteria, we predicted the essential genes of the yeast *Schizosaccharomyces pombe* and a similar performance was achieved (AUC score of 0.84). However, prediction of *Schizosaccharomyces pombe* essential genes using a model trained with the 15 bacteria yielded 0.65.

Our method is better than most of the existing predictors which rely on sequence information, only, and is on a par with the state-of-the-art predictors using homology, network topology, and gene-expression data in addition to sequence features.

We believe that the information-theoretic features can be effectively used in other biological classification problems. For instance, in [51] sequence motifs and k-mers were used for categorization of microRNAs. Hence, in the future, we will use the information-theoretic features for other prediction problems including microRNA detection.

## Additional file

Additional file 1 Feature selection and detailed results. **TableS1** provides an insight into the contribution of the different features. Detailed prediction results using various performance measures are provided in the other tables. (XLSX 78 kb)
